# GINI: From ISH Images to Gene Interaction Networks

**DOI:** 10.1371/journal.pcbi.1003227

**Published:** 2013-10-10

**Authors:** Kriti Puniyani, Eric P. Xing

**Affiliations:** School of Computer Science, Carnegie Mellon University, Pittsburgh, Pennsylvania, United States of America; Bar Ilan University, Israel

## Abstract

Accurate inference of molecular and functional interactions among genes, especially in multicellular organisms such as Drosophila, often requires statistical analysis of correlations not only between the magnitudes of gene expressions, but also between their temporal-spatial patterns. The ISH (*in-situ-hybridization*)-based gene expression micro-imaging technology offers an effective approach to perform large-scale spatial-temporal profiling of whole-body mRNA abundance. However, analytical tools for discovering gene interactions from such data remain an open challenge due to various reasons, including difficulties in extracting canonical representations of gene activities from images, and in inference of statistically meaningful networks from such representations. In this paper, we present GINI, a machine learning system for inferring gene interaction networks from Drosophila embryonic ISH images. GINI builds on a computer-vision-inspired vector-space representation of the spatial pattern of gene expression in ISH images, enabled by our recently developed 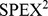 system; and a new multi-instance-kernel algorithm that learns a sparse Markov network model, in which, every gene (i.e., node) in the network is represented by a vector-valued spatial pattern rather than a scalar-valued gene intensity as in conventional approaches such as a Gaussian graphical model. By capturing the notion of spatial similarity of gene expression, and at the same time properly taking into account the presence of multiple images per gene via multi-instance kernels, GINI is well-positioned to infer statistically sound, and biologically meaningful gene interaction networks from image data. Using both synthetic data and a small manually curated data set, we demonstrate the effectiveness of our approach in network building. Furthermore, we report results on a large publicly available collection of Drosophila embryonic ISH images from the Berkeley Drosophila Genome Project, where GINI makes novel and interesting predictions of gene interactions. Software for GINI is available at http://sailing.cs.cmu.edu/Drosophila_ISH_images/

## Author Summary

As high-throughput technologies for molecular abundance profiling are becoming more inexpensive and accessible, computational inference of gene interaction networks from such data based on well-founded statistical principles is imperative to advance the understanding of regulatory mechanisms in various biological systems. Reverse engineering of gene networks has traditionally relied on analysis of whole-genome microarray data; here we present a new method, GINI, to infer gene networks from ISH images, thereby enabling exploration of spatial characteristics of gene expressions for network inference. Our method generates a Markov network, which encapsulates globally meaningful statistical-dependencies from vector-valued gene spatial patterns. In other words, we advance the state-of-art in both the usage of richer forms of expression data, and the employment of principled statistical methodology for sound network inference on such new form of data. Our results show that analyzing the spatial distribution of gene expression enables us to capture information not available from microarray data. Such an analysis is especially important in analyzing genes involved in embryonic development of Drosophila to reveal specific spatial patterning that determines the development of the 14 segments of the adult fly.

## Introduction

In multicellular organisms such as the metazoans, many important biological processes such as development and differentiation depend fundamentally on the spatial and temporal control of gene expression [1], [2]. To date, the molecular basis and regulatory circuitry underlying metazoan gene regulation remains largely unknown. Numerous statistical or algorithmic approaches have been attempted to infer “networks” of regulatory elements from high-throughput experimental data, based on various computational techniques like Bayesian networks [3]–[5], undirected Gaussian graphical models [6], [7], graph mining [8], ordinary differential equations [9], partial correlations [10], and others. Comparisons of different methods used for reverse engineering gene networks have been performed [11], [12], and predictions made by automatically learned gene networks have been experimentally validated [13], [14], thus increasing the credibility of such approaches.

This progress notwithstanding, a key deficiency of existing approaches is that they rely almost exclusively on univariate characteristics of gene states, such as a continuous-valued abundance measurement from microarray, or a binary on/off status derived from discretization of microarray data. However, microarray profiling of mRNA abundance can often be ill-suited for spatial-temporal analysis of gene expressions in multicellular organisms such as Drosophila, or in tissues/organs with natural or pathological progressions, because it captures only the “average” pattern of a sample. For any sample of interesting heterogeneous cell populations, the averaging operation would cause severe information loss and inaccuracy in downstream analysis (see Figure 1 in [15] for an intuitive illustration of how two genes with completely different spatial patterns over time yield near identical “average” temporal patterns.)

**Figure 1:**
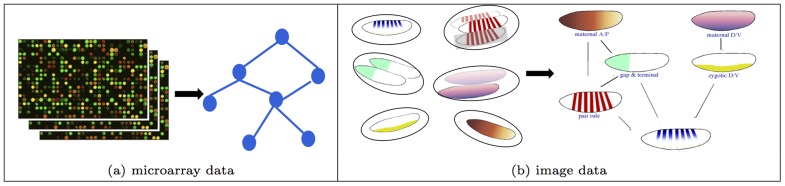
ISH analysis is more challenging than microarray analysis. (a) Univariate measurements taken simultaneously for all genes simplifies gene network inference from microarray data. (b) GINI extends such analysis to inferring a network from bags of images per gene.

Recent advancements in image-based genome-scale profiling technology such as whole-body mRNA abundance micro-imaging via *in situ hybridization* (ISH) have begun to reveal a more holistic view of the activities and functions of genes in rich spatial-temporal contexts. ISH has been used to characterize whole genome expression patterns for different species such as Drosophila embryos [16], [17], C. elegans [18], and adult mouse brain [19], and at smaller scales for Arabidopsis flowers [20], testicular germ cell tumors [21], and others. The availability of this form of gene expression data calls for development of next-generation image analysis systems to facilitate not only efficient pattern mining such as image clustering or retrieval, but also in-depth reasoning of complex spatial-temporal relationships between gene expression patterns, which will be essential for functional genomics and regulatory network inference in higher organisms. In this paper, we focus on a particularly interesting, but previously unaddressed challenge along this direction: inferring a statistically sound gene network from gene expression micro-imaging data, in the same sense of inferring a gene network from microarray data as widely studied in the literature. Analyzing ISH data allows us to infer a network by computing similarities in the spatial distributions of gene expressions in Drosophila embryo. Another important source of information is the temporal changes of the spatial distributions of genes, which could reveal how a gene regulation network evolves over time during dynamic biological processes such as embryogenesis [22]. We will defer the spatio-temporal network building based on time series of ISH data for future work as it requires the technique developed in this paper as a building block.

A major motivation of our work is the extensive imagery documentation of all the genes expressed during Drosophila embryogenesis via ISH imaging by the Berkeley Drosophila Genome Project (BDGP) [16]. BDGP is an ongoing effort to determine gene expression patterns during embryogenesis for Drosophila genes. In February 2013, the data contained more than 110,000 ISH images capturing the expression pattern of 7516 genes. Each image is annotated with time information, indicating the development of the embryo in six development stage ranges. Each image documents the gene expression pattern of a single gene in an embryo. Most images have a single embryo, however some images capture partial views of the embryo, others have overlapping or touching embryos. This is an extremely interesting but difficult dataset that reveals unprecedented details of gene activities during metazoan embryogenesis, but at the same time posts large unanswered challenges on methodologies for systematic and principled analysis. Specifically, we recognized the following main challenges that are unique to micro-imaging data versus the classical microarray data, which must be properly addressed before a genome-scale gene network can be derived from such data.

**Representation and quantification of gene activities:** Unlike microarrays, which represent gene activity with a univariate state or magnitude, images provide high-dimensional information for every gene, and it remains an open problem in computer vision research to extract meaningful features from the ISH images that are suitable for comparing activities of different genes and other genome-wide analysis [15], [23].
**Multi-variate measurement:** Even after one can standardize the imagery-records of the expression of a gene at a particular time point by a 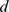-dimensional vector, where 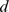 are the number of features extracted from the image, a proper metric must be defined to quantify distances between them.
**Condition alignment:** Images for different genes are typically taken under non-identical conditions (e.g., time, temperature, etc.), whereas a microarray is a snapshot of multiple genes under the same condition. This affects how signals are normalized across genes before they can be compared.
**Sample imbalance:** Different genes typically have different number of image records, i.e., for gene 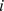 and 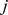, their corresponding measurements can be in the form of two bags of different sizes. It is not clear how to define distance or correlation between bags of images of different sizes. One simple solution to this problem is to randomly sample a single image from each gene. However, throwing away images fails to capture the natural variation observed in gene expression patterns for some genes. Further, if noise in the expression patterns has not been removed correctly in the feature extraction step, leveraging the existence of multiple images per gene can lead to reduced noise, and improved performance.
**Sparsity and statistical interpretability:** The interaction network proposed must be sparse and statistically meaningful, since we expect that a small fraction of all possible interactions are actually present in a single organism, and such interactions must reveal globally consistent conditional-independence relationships between genes, which is not possible in a simple pairwise-correlation graph.

There has been some earlier work on automatic annotation of ISH images with annotation terms [24], [25], clustering of gene expressions [17], determination of the development stage of embryos [26], etc., some of which have been applied on the BDGP dataset. In this paper, we propose a machine learning system to infer gene interaction networks from spatial similarity of gene expressions captured via ISH images. The system is called GINI, for Gene Interaction Network from Images. With such a system, we were able to address satisfactorily the challenges mentioned above, and systematically performed a genome-scale network learning and analysis on the BDGP dataset.

### Overview of the GINI approach

**Figure 2:**
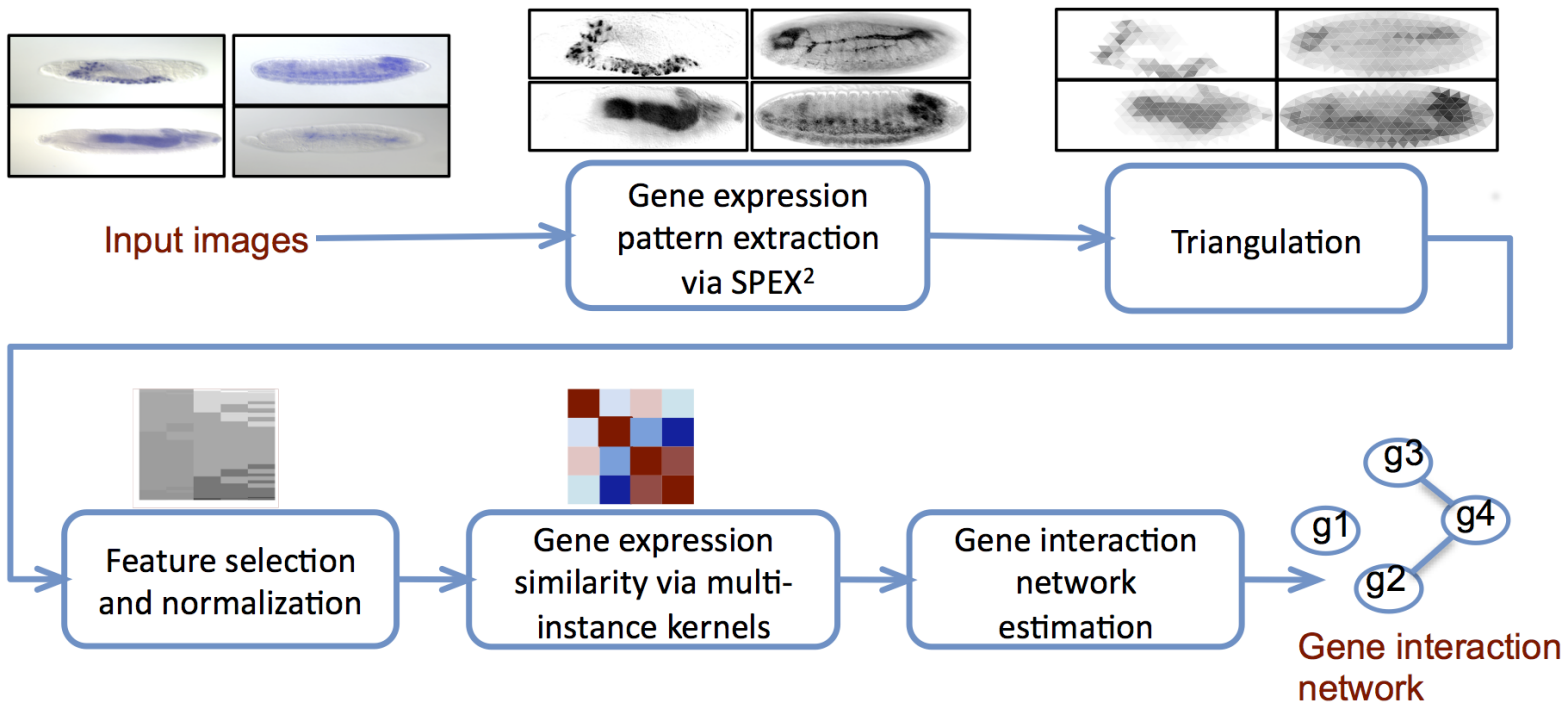
GINI schematic. The schematic shows an outline of the overall system to reverse engineer gene networks from ISH data. Sample output of each step is shown on top of the box corresponding to that step.

GINI first extracts the gene expression pattern from each image using a computer version driven image analysis pipeline 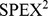 [15]. These expression patterns are spatially aligned and normalized to enable spatial comparison of gene expression across multiple images. Next, the expression patterns are represented by suitable standardized features through a process called “triangulation”, followed by feature normalization and selection. Since each gene may have a different number of images in the data, each gene can now be represented by a “bag” or a set of feature vectors - one feature vector per image. Thus, our task is to estimate the gene network, given bags of images per gene (Figure 1). We cast the problem of estimating a gene interaction network as the task of estimating the graph structure 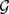 of a Markov random field (MRF) over the genes. The underlying graph encodes conditional independence assumptions between the genes, that is, two genes are said to not interact in the network if their gene expressions are conditionally independent of each other, conditioned on the expression of all other genes in the network. We employ multi-instance kernel technique using different order statistics to compute similarity between bags of images. We then estimate a sparse network of gene interactions by modeling the data as a multi-variate multi-attribute Gaussian, and estimating the sparse inverse covariance matrix of the model. A schematic diagram of the system pipeline can be seen in Figure 2.

### Contributions

GINI is a bioimage informatics system based on a computer vision pipeline for ISH micro-image processing and a statistical learning algorithm for network inference. The main contributions of this work are summarized below.

First, the image analytic pipeline used by GINI offers a rigorous and universal approach to extract a standardized representation of spatial patterns of gene expressions. Comparing to the popular SIFT features [24], which is based on detecting interest points with heavy assumptions on object shape, texture, and other physical properties originally meant for natural objects, our approach is more suitable for ISH staining in Drosophila embryos which do not resemble natural objects and require preservation of overall spatial shape and overall intensity information in a canonical way for intra-gene normalization and inter-gene comparison.

Second, GINI infers a network that enjoys the Markov network property: it gives globally consistent conditional-independency interpretation for every edge, and therefore is biologically more meaningful. It is known that marginal correlation (as often used in estimating an *ad hoc* network), which is computed for every gene-pair in isolation (i.e., ignoring all other genes in the system), confounds direct and indirect dependencies, and could result in a clique-like dense graph or subgraph among genes that are not directly dependent, but have a long-distance interaction. Studying conditional independencies in a network allows us to predict interactions between a pair of genes in the context of other genes, allowing a distinction to be made between direct and indirect relationships between the genes, and reducing false positives.

Third, our formulation based on Gaussian Markov random field and multi-instance kernel for the GINI network is convex, hence the globally optimal estimator of the network is computed, no approximations are involved. Furthermore, under suitable conditions, our graphical model learning algorithm is sparsistent, i.e. as the amount of available data increases, the algorithm is statistically guaranteed to predict the correct interactions between the genes. While Bach et.al. [27] have previously proposed learning the structure of graphical models from data using Mercer kernels, their approach is based on a non-convex local greedy search to find edges in the graph. Our approach represents the first work that uses Mercer kernels and Gaussian Graphical Models to predict kernelized graphical models using a convex formulation.

Finally, with the GINI system, we were able to systematically perform a genome-scale network learning and analysis of the genes expressed during 2 time points of Drosophila embryogenesis recorded by ISH imaging from BDGP [16]. In both time points, we find that the GINI networks are modular and scale free, which are properties predicted to hold true in gene interaction networks. Further, different regions of the networks are enriched for spatial annotations, thus GINI is able to cluster spatially similar genes. The hubs of the networks, i.e., the genes with the largest number of predicted interactions are functionally enriched for important cellular functions. We demonstrate that the networks predicted by analyzing microarray data does not have either spatial or functional enrichment, thus these results could not have been obtained by analyzing microarray data.

To the best of our knowledge, GINI represents one of the first efforts to reverse engineering gene networks from ISH image data. In both extensive simulation studies and empirical biological analysis, we demonstrate the effectiveness of GINI in predicting networks, and show that the statistical assumptions behind GINI are reasonable, and the biological analysis enabled by GINI merits close examination and further exploration.

## Methods

We begin by introducing the key algorithmic innovations needed to compute the gene network from the ISH images, assuming that each gene has a bag of images, with the images processed to be represented by informative and canonical feature vectors. This is followed by a discussion on the image processing procedures needed to extract informative features from the images.

### Network inference from “one image per gene” ISH data

We first show how GINI estimates a gene network, when each gene has only one image. The next subsection extends the GINI algorithm to deal with multiple images per gene.

Let 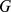 denote the set of 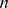 genes being studied, so that 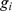 is the 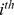 gene, where 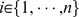, and 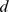 is the number of features extracted per image. Each feature represents the gene expression in a spatial location of the embryo.

Note that algorithms that analyze microarray data typically treat samples drawn from different time points as independent samples [28], even though expressions of the same gene across time is expected to be auto correlated. We similarly assume that the different spatial features are independent of each other. The spatial independence assumption has also been implicitly made by [29], [30] while modeling transcription networks in Drosophila embryos. In the results section, we use simulated data to demonstrate that this assumption does not affect the accuracy of the algorithm significantly.

By modeling the gene interactions as invariant across the spatial locations in the embryo, we can assume that each feature is independently and identically drawn (i.i.d.) from the same distribution. Inferring gene interactions is then equivalent to modeling the dependence between the expression values of different genes at the same spatial location. Expression of the 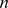 genes in each spatial location is assumed to be drawn from some (multi-variate) distribution, independent of all other spatial locations. Each spatial feature 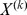 (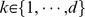) may be modeled as a vector of length 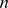, with 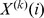 capturing the expression value of the 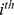 gene in this location 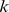. This gives us 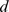 independent samples with which the parameters of the underlying distribution may be learned. Formally, let each spatial location be drawn independently from a multi-variate Gaussian 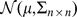, where 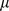 is the mean vector, and 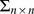 is the positive semi-definite covariance matrix between the genes.

In a multivariate Gaussian distribution, the 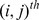 entry of the inverse covariance matrix 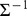 is zero if and only if the corresponding genes are conditionally independent given the rest of the graph. Thus, the non-zero entries of the inverse covariance matrix correspond to edges in the corresponding Gaussian Markov random field, giving rise to the gene interaction network. The Gaussian Markov random field is also known as a Gaussian graphical model (GGM) [31]. Since we expect a small number of interactions per gene, the estimated graph must be sparse, i.e. the number of non-zero entries of the inverse covariance matrix must be small.

Thus, the gene interaction network may be estimated by learning a Gaussian distribution from the observed images, such that the inverse covariance matrix is sparse. The mean 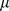 of the Gaussian is estimated by the observed sample mean,
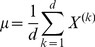
(1)


Then, the inverse covariance matrix 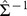 can be estimated by minimizing the negative log-likelihood of the data, over all possible positive semi-definite matrices. To enforce sparsity, the 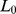 norm of 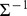, which counts the number of non-zero elements, is added to the negative log likelihood. Since optimizing the 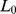 norm is non-convex and NP hard, the 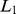 norm is used as a convex relaxation to the 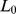 norm. The 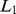 norm of a matrix is the sum of the absolute values of the elements of the matrix, and also enforces sparsity in the solution. Adding the 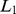 norm regularization also ensures that the minimizer of the objective function exists, and is well defined. Thus, our objective function is
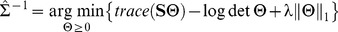
(2)where 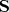 is the second moment matrix about the mean
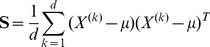
(3)

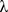
 is a tuning parameter, by which we determine the strength of the penalty. As we increase the value of 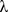, we increase the penalty on the absolute values of 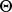, and hence, the graph induced by 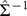 becomes more sparse. The edges in the graphical model are then estimated as
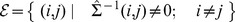
(4)


#### Optimization

The objective function defined in Equation 2 is convex, hence it can be solved by any convex optimization algorithm. Banerjee et. al. [32] formulated an 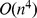 block coordinate descent method to solve it, where 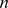 is the number of dimensions. Friedman et. al. [33] formulated each step of the block coordinate descent as a Lasso regression, and solved it in 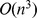 - they named their technique glasso. The glasso algorithm uses a series of 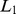 penalized regressions, called Lasso regressions [34]; and we use the glasso algorithm for efficient optimization of our objective function.

Note that Equation 2 is a function of data 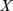 only through the sample covariance matrix 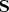, hence, we can replace the sample covariance matrix with a suitable similarity or kernel function. This is the key idea behind GINI's algorithm to deal with multiple images per gene, which we discuss in the next section.

### Network inference from “multiple images per gene” ISH data

Multiple images of the same gene at the same time point should have the same gene expression pattern. However, in practice, the expression patterns in different images may differ considerably, for three main reasons.

Firstly, there is a wide interval of time considered as a single time point while collecting such data. For instance, the BDGP data divides embryonic development into 6 time stages. The last stage 13–16 corresponds to development of the embryo 9.3 to 15 hours after fertilization, which represents more than a third of the time taken for embryonic development. Hence, the true gene expression pattern may be dynamic within the time period of a single development stage, and the gene expressions captured for the same gene at the same time may not look similar to each other. Secondly, we might expect that for any organism for which ISH data is collected, there will necessarily be some ambiguity in how the development stage of the organism is labeled by human annotators. Finally, noise in the expression patterns due to excessive staining, lighting conditions and similar other reasons will also be observed. For all of the above reasons, any network-learning algorithm should leverage the existence of multiple images per gene per time point in improving its estimates of gene similarity.

The problem of multiple images per gene is reminiscent of multi-instance learning [35], [36]. Multi-instance learning is a form of supervised learning, where instead of labeling each instance, a bag of instances is labeled. A popular solution to the multi-instance problem is to define a multi-instance kernel, that can compute the similarity between bags of instances. Let 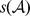 be a collection of order statistics of the set 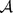, for example, mean, median, minimum, maximum etc. In 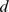 dimensions, 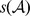 is computed on each dimension independently, to form a vector of order statistics. If we use 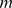 order statistics, then the length of 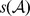 will be 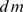. The similarity between gene 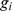 with a set of images 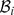 and gene 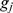 with images 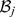 can then be computed as
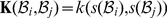
(5)where 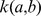 is an appropriate kernel function between vectors 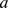 and 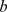. Such a kernel is called the statistic kernel.

The choice of the order statistics used in the kernel depends on the data collection procedure of the ISH. One concern in ISH data is that images may be overstained. In such a scenario, the median may be an appropriate choice of order statistic. If over-staining is not a concern, the maximum statistic may be more appropriate to ensure that information about presence of gene expression is not lost.

For the BDGP data, we use the covariance kernel 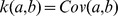, and the mean statistic 
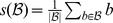
. The choice of using a single statistic to represent information from multiple images was due to the presence of noisy images in the data set. Thus,
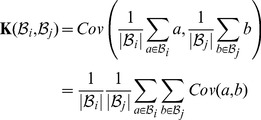
(6)


Thus, our choice of kernel is equivalent to computing the mean similarity of all pairs of images in bags 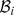 and 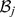. This specific kernel is also known as the normalized set kernel, and has been shown to perform very well in multi-instance classification [37].

Any kernel function may be written as the dot product in some higher dimensional feature space, i.e. 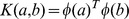 [38]. Hence, if we assume that the data is drawn from a distribution such that 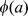 is a zero-mean Gaussian, we can learn the gene interaction network by treating 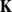 as the sample covariance matrix. Since estimating the inverse covariance matrix by solving equation 2 requires only the sample covariance matrix 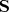 and not the data itself, we can kernelize it by using the kernel matrix 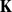 defined in equation 6 as the required sample covariance matrix. Thus, the objective function is
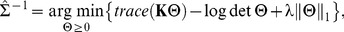
(7)which can be solved as discussed in the previous section.

#### Consistency of the estimate

Given samples 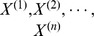 drawn from a Gaussian distribution, it can be shown that the objective function in Equation 2 leads to a consistent solution, with a suitable choice of 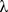 [32]. That is, the estimator 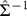 converges in probability to the true inverse covariance matrix 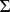.

GINI however does not work with samples from a Gaussian distribution, but directly with a multi-instance kernel 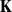. By definition, any kernel 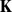 can be represented as an inner product in some feature space 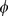, i.e. 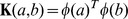. For the multi-instance statistic kernel, 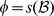, that is, the feature space is defined by the order statistics computed over bag 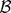. Since the order statistics for image data is bounded between 0 and 255, 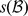 is a bounded random variable. Hence the distribution of 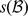 is sub-Gaussian. For sub-Gaussian distributions, Ravikumar et. al. [39] show that the penalized maximum likelihood estimator defined in Equation 7 is sparsistent, i.e. as the amount of data increases, the probability of identifying incorrect edges goes to zero. Thus, the kernelized estimator defined by GINI is sparsistent.

Thus, the GINI algorithm predicts the gene interaction network in two steps: in the first step, the similarity between different genes is computed using a multi-instance kernel. In the next step, a sparse interaction network is learned from the similarity matrix by solving Equation 7, and predicting edges corresponding to the non-zeros of the non-diagonal entries of the estimated 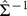. The next subsections describe the feature extraction, representation, and normalization process used to obtain suitable features from images that can be input into GINI.

### Image processing

We convert the ISH images into canonical feature vectors suitable for analysis by our algorithm described above in a three-step manner. First, the precise expression pattern found in each image is extracted and aligned spatially to make all images spatially comparable. Next, each image is represented by a feature vector using Delaunay triangulation. Finally, features are normalized and feature selection is performed to extract meaningful features, that can be then used to compute the multi-set kernels to obtain gene similarity and learn the gene network.

#### Feature extraction via 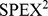


ISH images are taken under diverse lighting conditions, and may suffer from poor quality staining/washing. A good feature extraction system must remove these effects, controlling for position, orientation etc. of the embryo and extract a precise gene expression pattern from the ISH images. In previous work [15], we developed 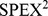, an automatic system for embryonic ISH image feature extraction. 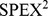 registers each Drosophila ISH image by first extracting the embryo (foreground) from the image, using edge filters and image analysis techniques. Next, the alignment, size, shape and orientation of the embryo is determined, and normalized to a standardized ellipse. 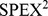 also does automatic error detection and correction, rejecting images where the gene expression extraction process may have introduced errors, and also rejecting partial embryos, multiple embryos physically touching each other, and excessively dried or otherwise mishandled embryos. Next, the expression stain is extracted from the standardized embryo using a novel algorithm that maximizes the contrast between the stained and unstained regions of the embryo. Finally, an image segmentation algorithm using Markov random fields is defined to extract only the regions that have gene expression. Thus, a concise and high-fidelity gene expression pattern is extracted from the ISH image.

#### Feature representation via Delaunay triangulation

While 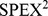 makes the images of different genes alignable spatially, and therefore directly comparable, the expression patterns must still be converted into an appropriate feature representation. One commonly used method for feature representation is to use the SIFT feature descriptor [40] in either a grid of points spaced uniformly through the image, with principal component analysis (PCA) used for dimensionality reduction [15], or via interest point detection and codebook generation [24]. Such techniques work well for supervised tasks like image annotation where a weight can be learned for each direction computed by PCA or for each codeword in the generated codebook. However, for unsupervised tasks, where weights cannot be learned, we wish to extract features that explicitly take into account the spatial distribution of the gene expression. A pixel level feature representation on the other hand, allows us to capture spatial information, but has high redundancy due to the correlation between neighboring pixels.

To reduce redundancy while capturing spatial gene expression information, we choose to overlay a fixed triangular mesh on top of the standardized embryo. The gene expression pattern for each image may then be represented as the median gene expression present in each triangle in this mesh. A mesh of 311 equilateral triangles was produced by using the Delaunay triangulation algorithm [41], and aligning the mesh to the standardized embryo, as described in [23]. Each image can then be represented as a feature vector of length 311, with each feature representing the median gene expression expressed in a specific location on the embryo, which is fixed across all images. For example, triangle 1 may correspond to the head in all images, and so on. Modeling the spatial locations in a lower dimensional space via triangulation helps in approximating the independence assumption made in the GINI algorithm, analogous to using coarse time definitions while making microarray measurements.

**Figure 3:**
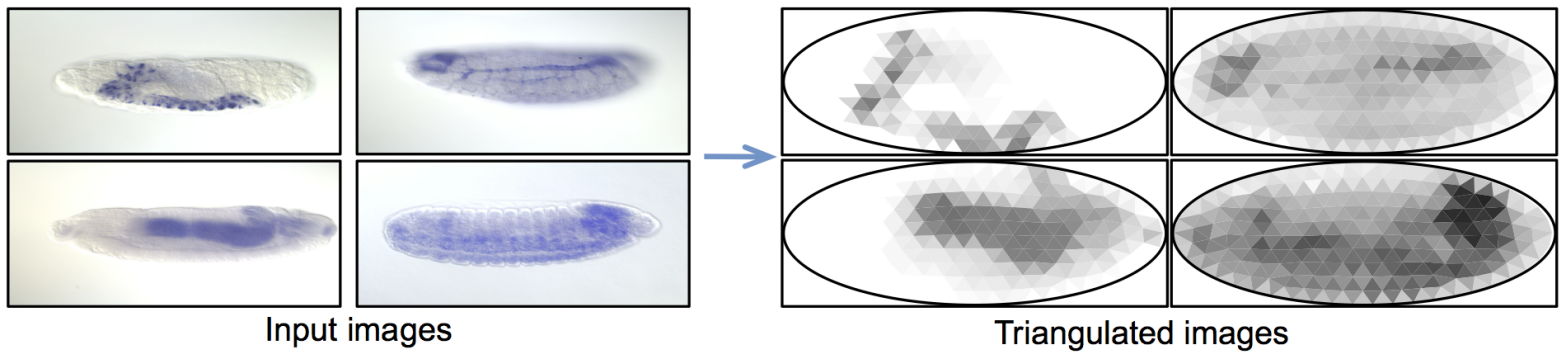
Triangulation. Examples of how ISH images are converted into low-dimensional triangulated representations, for efficient feature representation.

Figure 3 shows examples of ISH images converted into the triangulated gene expression patterns. As can be seen, triangulating the 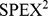 output captures the key features of the gene expression location and strengths. Thus, triangulation enables reducing the dimensionality of the feature space, while retaining explicit spatial information about the gene expression, which other dimensionality reduction techniques would not be able to capture.

#### Feature processing

The feature vectors extracted by triangulating the expression patterns are not normalized, hence, we need to adjust the signal obtained from different images to a common scale. The set of triangulated features may also contain uninformative features that may add a bias if used directly to compute the multi-set kernel. Further, the gene network analysis should only consider genes with informative expression patterns that have non-trivial spatial expression in the data. Hence, we need to further process the features to select informative features and genes, and normalize the features in an appropriate manner.

#### Feature normalization

Unlike in microarray data, the currently available ISH data does not measure the signal related to nonspecific binding of the probe for each image, hence the background correction of intensities cannot be image specific. Each gene expression pattern is normalized to have its expression values(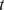) lie between 0 and 255 (the minimum and maximum color value). The feature value is then computed as the logarithm of the expression value : 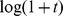.

#### Feature & gene selection

A large percentage of the ISH images have no stain, or ubiquitous staining. In the BDGP data, 55% of the genes have at least one image, in at least one time point, with no stain. Since no information may be inferred from such data, these images must be removed from analysis. This can be achieved by removing expression patterns having variance below a threshold (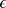, usually 0.1).

Additionally, features that have low variance in the data set are capturing no information about the gene expression variation across multiple genes. Hence, they must be removed from the analysis as well. Since removing images from the analysis affects the feature variance and vice-versa, we alternate removing features and images with low variance, until both feature variance and image variance is greater than the threshold. This is described formally in Algorithm 1.

**Algorithm 1. Algorithm Outlining Feature Normalization and Processing.**

**Input**: triangulated features for 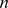 images : 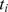, where 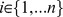;

  variance threshold 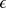

**Output**: normalized and processed features in matrix 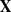

**for** *image* 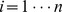 **do**

 

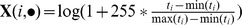
;

**end**

**while** 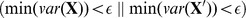 **do**

 

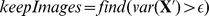
;

 

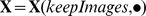
;

 

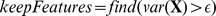
;

 

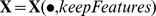
;

**end**

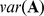
 for matrix 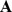 returns a vector containing the variance of each column of 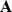; 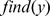 returns the indices of the non-zero elements of vector 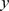, and 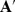 is the transpose of matrix 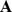.

### Summary of the GINI system

Putting everything together, we conclude the method section with a summary of the GINI system for network inference from ISH images. Each ISH image is converted into a standardized expression pattern using 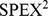, and then triangulated to extract a low-dimensional spatial feature vector. Next, feature values are normalized, uninformative features are removed, and genes with insufficient information available are rejected. Finally, the multi-set kernel is used to compute the similarity between the bags of image vectors available for each gene, and the gene network is estimated using Equation 7. The algorithm is summarized in Algorithm 2.

**Algorithm 2. The Final GINI Algorithm to Obtain the Gene Network from ISH Images**

**Input:** Embryonic ISH images for 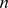 genes;

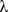
 - tuning parameter to control sparsity

**Output**: Predicted gene network for the 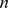 genes

**for** *gene* 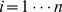 **do**

 // feature extraction

 

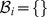
;

 
**for** *each image* 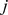 of gene 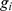 **do**

  Extract expression patterns from image 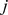 using   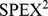;

  

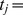
triangulate expression pattern of image 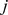;

  // feature normalization

  

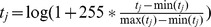
;

  

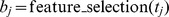
;

  

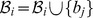
;

 
**end**

 // 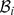 is now the set of all normalized features of the  images of gene 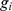

**end**

// Define the multi-instance kernel

**for** *gene* 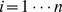 **do**

 
**for** *gene* 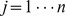 **do**

  

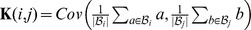

 
**end**

**end**

// Run glasso using kernel 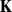

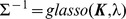
;

Predicted edges in the network: 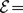
non-zeros in the non-diagonal elements of 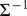;

#### Computational complexity

We assume that the number of images per gene is small and bounded by a constant, and hence the total number of images is 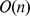, where 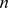 is the number of genes. Then, given the triangulated features of all images, feature and gene selection takes time 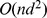 and 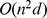 to compute the correlation matrix in feature and gene space respectively. Computing the kernel requires 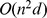 time, and finally, the computational complexity of minimizing the log-det divergence is known to be 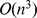. The overall computational complexity is then 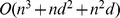. Assuming 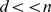, the complexity may be assumed to be 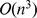. The implementation is efficient, and computes a gene network for 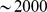 genes in a few minutes on an Intel Core-2 CPU with 2 GB memory.

## Results

We first demonstrate that the independence and Gaussian assumptions are reasonable for ISH data, and that GINI explains the ISH data well, with small fitting errors, and no bias in the residues. Next, we show the performance on a small subset of 12 images for 6 genes to verify that the network predicted by GINI is reasonable. We then run GINI on two datasets of ISH images from 2 time points in the BDGP data, and study the networks. We find the networks are modular and scale free as expected. Furthermore, different regions of the networks are enriched for spatial annotations, and the hubs of the networks are functionally enriched for important cellular functions. Finally, we demonstrate that these results could not have been obtained by analyzing microarray data.

### Validation of the GINI assumptions: Independent spatial data

GINI assumes that the gene expression in each triangle can be assumed to be independently drawn from a multi-variate Gaussian. However, the true gene expression in adjacent spatial locations is correlated and not independent. To verify that this dependence of adjacent samples does not affect the accuracy of the estimated network, we simulate synthetic data where the underlying network is known, but the data points are not independent of each other, and test whether GINI can recover the correct network in such a scenario. The data samples depend on each other via a parameter 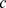 that captures degree of dependence between data samples. When 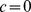, all data samples are drawn i.i.d. from the known distribution. As 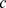 increases, data samples are drawn from the same distribution, but they depend on the adjacent samples.

#### Data generation

For 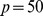 dimensions, the true inverse covariance matrix was constructed by using the AR(1) model from [42]. That is, 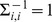, and 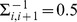, with all other elements being zero. Dependent samples are generated from a zero mean Gaussian having the above known inverse covariance matrix, as explained below.

Let 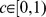 be the fractional overlap between adjacent samples. The first sample is sampled independently from the above specified Gaussian. The 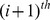 sample is generated from the 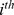 sample as follows. Pick 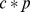 random features 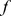, and copy the value of the previous sample for these features : 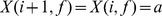. Now, 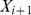 can be partitioned into the “known” 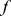 features and the remaining 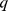 features which still need to be sampled, conditioned on 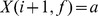. If we partition 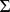 as
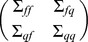
then 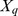 conditioned on 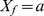 can be shown to be Gaussian with mean 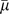 and covariance 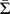, which can be computed as below, and 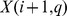 can be sampled from it.
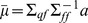
(8)

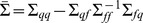
(9)


We ran two experiments. In the first, a fixed number of samples(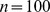) were used to learn the network. In the second, as 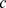 increases, more samples (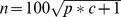) are available for learning the network. In both experiments, for each 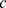 value, we randomly sample data points 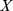 using the method outlined above, estimate the 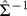 matrix, and compare it to the known 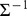 matrix, to compute precision and recall. Results are averaged over 50 runs of the experiment.

**Figure 4:**
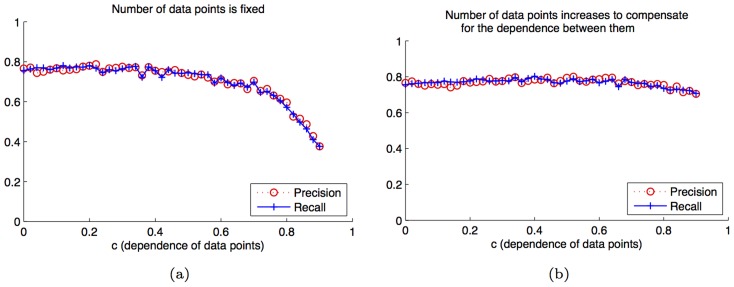
GINI assumptions are reasonable. Even if the data are not independent draws from the Gaussian, the network can still be learned with high precision and recall. (a) For a fixed number of data points, as 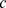 increases beyond 0.5, the precision and recall reduces. (b) If we allow the number of data points 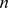 to increase as 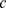 increases, the precision and accuracy of the method is not affected. The standard deviation at each point in both results is approximately 0.09.

Figure 4(a) shows that as 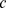 increases, the precision (fraction of correct interactions among all inferred ones) and recall (fraction of correct interaction among all true interactions) stay constant for small values for 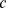. Only when the amount of dependence increases beyond half, do we see a small reduction in accuracy. Thus, we can conclude that even if there is a large spatial dependence in gene expression, the result is equivalent to a slight reduction in performance. Futher, in Figure 4(b), we see that if we can increase the number of data points as we increase 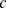, the performance remains the same as using i.i.d. data.

### GINI explains the ISH data well

For a high-dimensional distribution, it is not feasible to test if the data is truly Gaussian. However, a consequence of Gaussianity is that for each gene, the gene expression can be expressed as a weighted linear sum of the expression values of a few other genes, which form the edges of the network. To test if this assumption holds true in ISH data, for each gene, we fit a linear regression between the gene and its neighbors found by GINI and look at the absolute value of the error i.e. the mean absolute difference between the predicted and the known gene expression. When the maximum expression value is 1, for more than 90% of the genes we looked at, the absolute error was less than 0.02; 99.5% of all genes had absolute error less than 0.05, confirming that the GINI generative model explains the ISH data.

**Figure 5:**
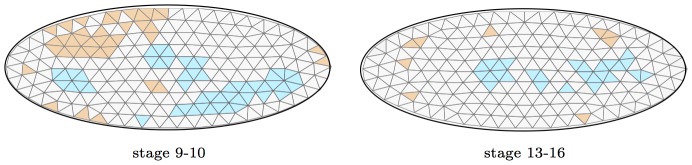
GINI error analysis. Locations where gene expression cannot be predicted easily. Red color indicates that the true gene expression was higher than predicted by the regression, while blue indicates that the true gene expression was lower than predicted by the regression. Note that since the difference in the true and predicted gene expressions is very small, the mean residue values were multiplied by 10 to improve the contrast of the image for visualization purposes. Thus, there is no systematic bias in the spatial locations where expression is hard to predict.

We also confirm that the prediction error is not systematic with respect to the spatial location. For each gene, we compute the prediction error (residue) when the gene is predicted by regressing it on its neighbors. For each spatial location, we plot the mean residue at that location for all genes. As can be seen in Figure 5, there is no systematic bias in the spatial positions that are hardest to predict for any gene.

### Network on limited data

**Figure 6:**
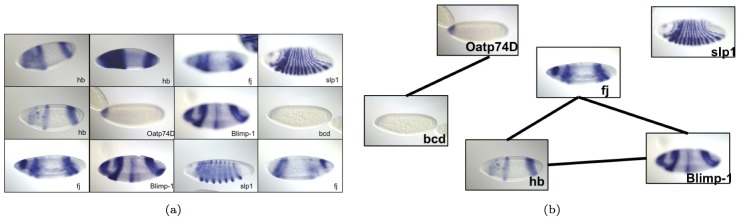
Example network on small data. (a) 12 images input to the GINI system, and (b) network of genes learnt by it, with each gene represented by one image.

Before running our algorithm on a large sized dataset, we construct an artificial small data set to verify the results. We input 12 images, shown in Figure 6(a) from 6 genes to the GINI algorithm (each gene has 1–3 images in the data set). With 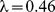, 4 edges are predicted in the network, shown in Figure 6(b). As can be seen, the three genes hunchback(*hb*), four-jointed(*fj*), and *Blimp-1*, which are expressed in the dorsal, ventral and procephalic ectoderm, are connected in a single cluster. Similarly, the genes organic anion transporting polypeptide 74D(*Oatp74D*) and bicoid(*bcd*) are connected by an edge, since both show expression in the foregut and the anterior endoderm. Finally, the expression of sloppy paired-1*(slp1)* was considered to be sufficiently different from the other genes, hence it is not connected to any other gene in the network.

Thus, the gene interaction network found by GINI can be verified to be reasonable for the above small data set.

### Network on the whole BDGP data

We now turn our attention to the ISH images from the Berkeley Drosophila Genome Project data set. We have obtained around 67400 ISH images of 3509 protein-coding genes from the BDGP data released in September 2009, captured at key development stages of embryonic development. Each image captures embryonic gene expression of a single gene using RNA in-situ hybridization. Each image was labeled manually with the age of the embryo, categorized into six distinct embryonic stages : 1–3, 4–6, 7–8, 9–10, 11–12, and 13–16. Genes are also annotated with ontology terms from a controlled vocabulary of around 295 terms, describing the unique embryonic structures in which gene expression is observed during the various stages of embryonic development. 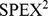 analyzes these image automatically, rejecting unsuitable images, to produce 51593 expression patterns of 3347 genes.

As proof of concept, we focus on images viewed from a lateral perspective from two development stage ranges of this data : 9–10 and 13–16. For the stage 9–10, we have 2869 expression patterns of 2609 genes, and for stage 13–16, we have 6350 expression patterns of 3258 genes. We extracted features as described in the methods section. For each development stage, we ran a separate analysis.

Using a 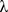 value of 0.775 for stage 9–10, we ran GINI and obtained a network having 258 genes, and 516 interactions (edges) between them. For the development stage 13–16, we used 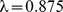, and obtained a network with 1202 genes and 3666 interactions between them. The 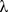 value was selected for each network by running GINI for 21 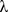 values between 0.5 and 1, and picking a value such that the mean-degree for the network is reasonable (approximately 2–3) - see Supplementary Figure S1 for a plot that shows how the number of edges in the network decreases as 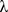 increases.

Some of the interactions predicted by GINI have already been reported in the literature. For example, in the network for stage 9–10, GINI predicts that DCP-1 (CG5370), an effector caspase which is involved in apoptosis, will interact with the *thread* gene (CG12284), a known inhibitor of apoptosis protein [43]. GINI also predicts that *Snf5- related 1*(CG1064) interacts with *echinoid* (CG12676), both of which are known to be involved in epidermis development, muscle organ development, as well as imaginal disc-derived wing vein morphogenesis. In the 13–16 development network, GINI predicts that the *capping protein beta* gene (CG17158) interacts with the *Glycogen phosphorylase* gene (CG7254), and *Tpc1* (CG6608) interacts with CG2812, which has been previously reported in [44].

The next five subsections do a detailed analysis of the 2 networks.

### Scale free network

**Figure 7:**
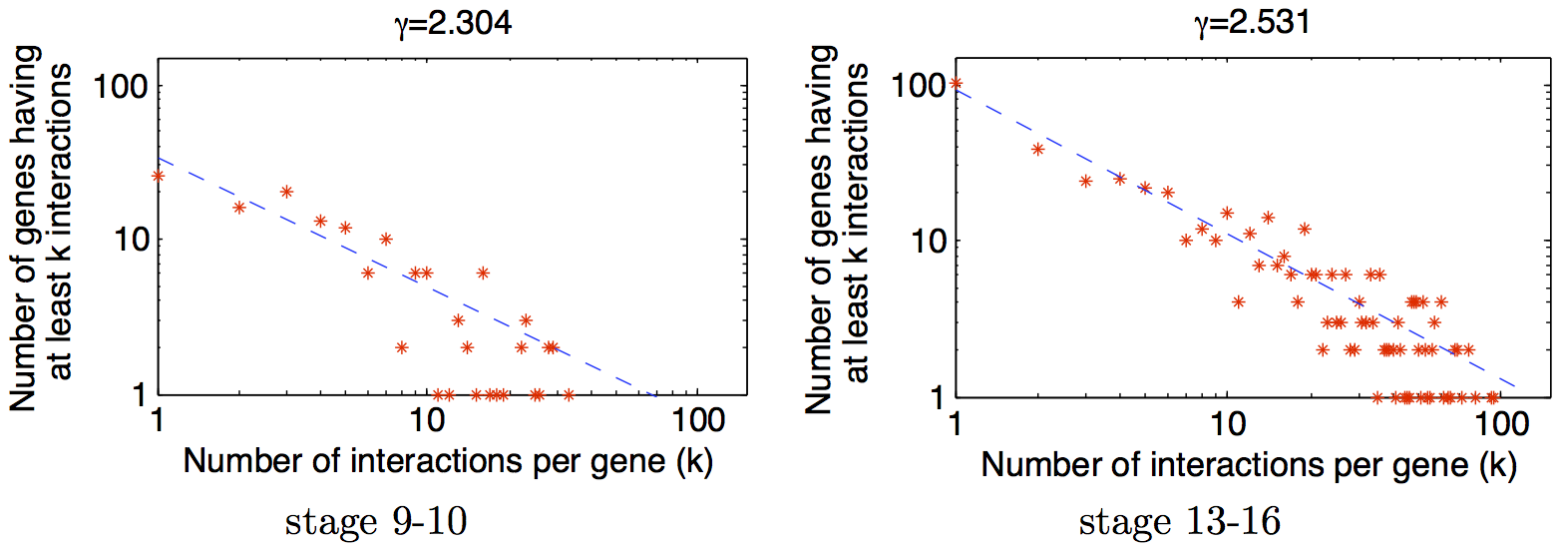
Scale-free network. Connectivity properties of the reconstructed network for time stage 9–10, and 13–16. The scale free nature of the plot can be observed for both networks. The plot for stage 9–10 has fewer points since the network constructed has fewer nodes and edges.

A network is said to be scale free if its degree distribution asymptotically follows a power law. That is, the fraction of genes 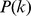 that have at least 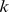 interactions with other genes is
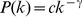
(10)where 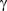 is the scale free parameter, and 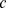 is the normalization constant. It has been hypothesized that gene regulatory networks are scale free [10]. We looked at the characteristic of our interaction networks by plotting the number of interactions per gene (Figure 7), and found that the networks found by GINI are scale free. The 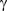 parameter obtained is 2.3 and 2.5 for the 9–10 and 13–16 networks respectively, which corresponds well to the values observed for a large variety of power law graphs. The scale free nature of the network was found to be independent of the 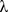 tuning parameter of the algorithm.

Unlike the gene regulatory network obtained for Human-B cells [10], we found that the scale-free nature of the gene network we obtain has a good fit, without observing a deviation from the expected at low connectivity values. However, this could be a side-effect of the larger number of genes they analyzed.

### The BDGP networks are modular

**Figure 8:**
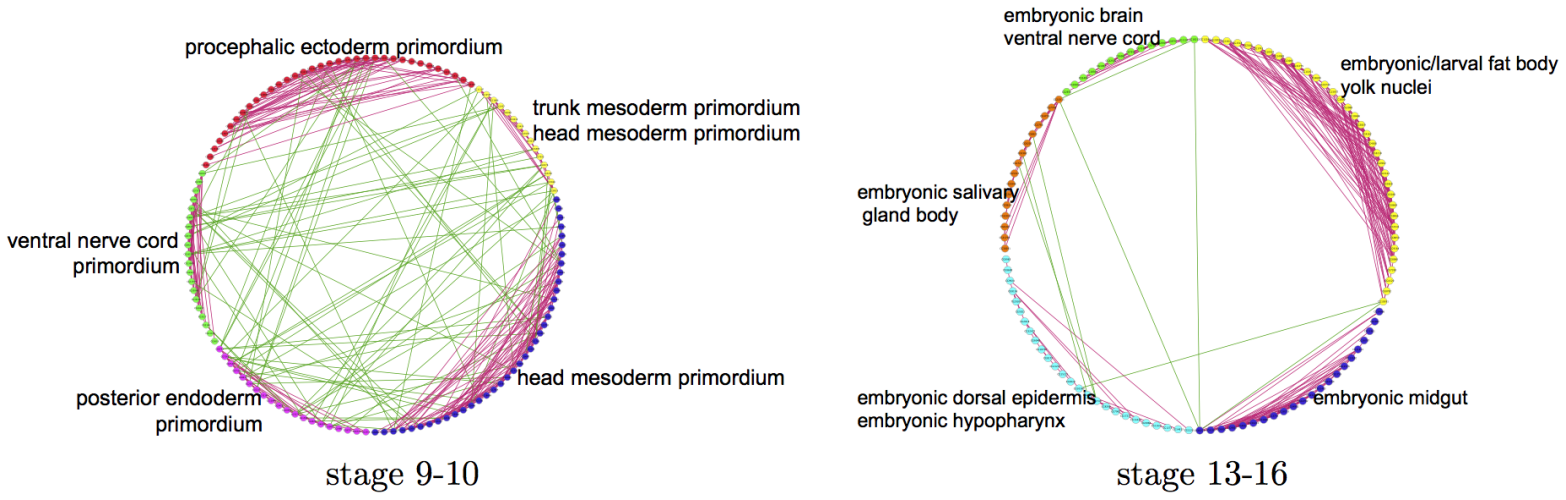
Modular network. A global view of the networks constructed by our algorithm for development stage 9–10, and 13–16, visualized for 5 of the 12 clusters in the network. The nodes of each cluster in the network are represented by different colors. Red edges are edges between nodes in the same cluster, while green edges are edges between nodes in different clusters. Each cluster is represented by one or two spatial annotation terms enriched in the cluster.

Using spectral clustering, we construct 12 regions or clusters within each network, and visualize the five biggest clusters of each of the networks in Figure 8. All 12 clusters in both networks are very well separated. The ratio of within-cluster edges to total number of edges is 70% and 87% for the 9–10 and 13–16 development stage networks respectively, indicating that the estimated networks are highly modular. From a biological perspective, different parts of gene networks may be responsible for different pathways or biological functional components of the cell, thus modularity is a good prediction for real interaction networks.

### Hub analysis

Given the scale-free nature of the network, a small number of the genes have a large number of interactions. We analyze the Gene Ontology functions of the genes having the largest number of interactions, i.e. the hubs of the network. The question we wish to address is: if we pick the top 5% of the genes having the maximum connectivity with other genes, what kind of functional enrichment do these genes have? Our background population is of the 2609 and 3258 genes for which we have at least one ISH image describing its expression for the 9–10 and 13–16 stages respectively. We use the hypergeometric test, with Bonferroni correction used to correct for multiple hypothesis tests [45]. As can be seen in Table 1, we observe enrichment of a wide variety of functions that are essential to cell growth and functioning, including metabolic processes, cellular respiration, transport of electrons and ions, protein modification, ribosome biogenesis etc.

**Table 1:** GO functional analysis for the gene hubs of the GINI network.

Stage	Gene Ontology term	Hub frequency	Genome frequency	P-value
9–10	cellular macromolecule metabolic process	57 of 119 genes, 47.9%	652 of 2575 genes, 25.3%	1.79e-05
	macromolecule metabolic process	62 of 119 genes, 52.1%	772 of 2575 genes, 30.0%	8.16e-05
	cell cycle	24 of 119 genes, 20.2%	174 of 2575 genes, 6.8%	0.00029
	primary metabolic process	67 of 119 genes, 56.3%	962 of 2575 genes, 37.4%	0.00559
	cell cycle phase	18 of 119 genes, 15.1%	127 of 2575 genes, 4.9%	0.00667
	cellular metabolic process	64 of 119 genes, 53.8%	910 of 2575 genes, 35.3%	0.00827
	mitotic cell cycle	18 of 119 genes, 15.1%	131 of 2575 genes, 5.1%	0.01039
	cell cycle process	19 of 119 genes, 16.0%	146 of 2575 genes, 5.7%	0.01313
	cellular process	90 of 119 genes, 75.6%	1508 of 2575 genes, 58.6%	0.01798
	macromolecule modification	20 of 119 genes, 16.8%	163 of 2575 genes, 6.3%	0.01888
	protein modification process	19 of 119 genes, 16.0%	155 of 2575 genes, 6.0%	0.03111
	nucleobase-containing compound metabolic process	39 of 119 genes, 32.8%	476 of 2575 genes, 18.5%	0.04665
13–16	energy derivation by oxidation of organic compounds	13 of 159 genes, 8.2%	47 of 3217 genes, 1.5%	0.00011
	cellular respiration	12 of 159 genes, 7.5%	43 of 3217 genes, 1.3%	0.00031
	generation of precursor metabolites and energy	14 of 159 genes, 8.8%	60 of 3217 genes, 1.9%	0.00039
	electron transport chain	10 of 159 genes, 6.3%	32 of 3217 genes, 1.0%	0.00093
	mitochondrial ATP synthesis coupled electron transport	9 of 159 genes, 5.7%	26 of 3217 genes, 0.8%	0.00121
	ATP synthesis coupled electron transport	9 of 159 genes, 5.7%	27 of 3217 genes, 0.8%	0.00174
	cellular process	116 of 159 genes, 73.0%	1808 of 3217 genes, 56.2%	0.00211
	respiratory electron transport chain	9 of 159 genes, 5.7%	28 of 3217 genes, 0.9%	0.00246
	oxidative phosphorylation	9 of 159 genes, 5.7%	29 of 3217 genes, 0.9%	0.00342
	ribosome biogenesis	7 of 159 genes, 4.4%	20 of 3217 genes, 0.6%	0.01640
	cellular metabolic process	78 of 159 genes, 49.1%	1091 of 3217 genes, 33.9%	0.01708
	mitochondrial electron transport, NADH to ubiquinone	6 of 159 genes, 3.8%	15 of 3217 genes, 0.5%	0.02661

GO functional analysis for the gene hubs of the networks learned for the two development stages by GINI. Both networks have hubs that are enriched for multiple important cellular functions.

**Figure 9:**
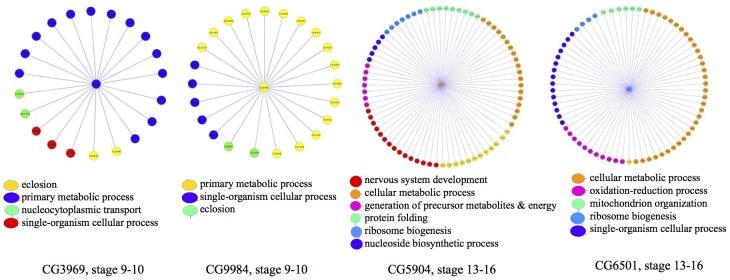
Hubs of the GINI network. A look into the neighborhoods of a few hubs from the GINI networks for stage 9–10 and 13–16. A few enriched GO groups are highlighted in the subnetworks as shown.

Next, we examine a few high-degree hubs in the two networks in detail, along with their neighborhood genes in the networks. Figure 9 shows the hub neighborhood for two genes in the 9–10 development stage network. CG3969 is a Activated Cdc42 kinase-like gene known to be involved in protein phosphorylation [46] and cell death [47], and CG9984 (*TH1*) is known to be involved in regulation of biosynthetic process [48] and nervous system development [49]. Both genes interact with many genes having functions related to the primary metabolic process, and single-organism cellular process. In stage 13–16, we examine the hub neighborhood of CG5904 and CG6501. The mitochondrial ribosomal protein CG5904 has been previously predicted to be a structural constituent of ribosome [50], and we find that it interacts with many genes involved in the ribosome biogenesis. Gene CG6501 (*Ns2*) has been previously predicted to be involved in phagocytosis, engulfment [51], and ribosome biogenesis [46]; CG6501's neighborhood has multiple genes that are also involved in ribosome biogenesis and single-organism cellular process.

### Enrichment of annotation terms

Each gene in the BDGP data has been labeled manually by annotations describing the spatial gene expression, using 295 annotation terms. We expect that since the gene interaction network is constructed via spatial similarity, genes that are connected to each other in the network will have similar spatial annotation terms.

**Figure 10:**
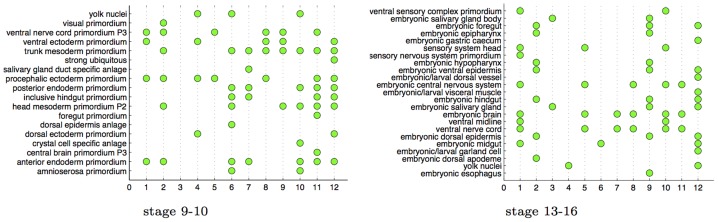
Spatial annotations. Enrichment analysis for clusters in the gene interaction networks found by GINI. A green dot indicates enrichment with a P-value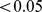.

To test this, we cluster the gene network using spectral clustering [52] into 12 clusters, and analyze the enrichment of each cluster for annotation terms using the hypergeometric test, with Bonferroni correction used to correct for multiple hypothesis tests. In the gene network for the 9–10 stage, 11 of the 12 clusters are enriched for 63 total annotation terms (Figure 10). The only cluster not showing any enrichment in the 9–10 stage network is also the smallest cluster, having only 4 genes. For example, in cluster 8, 92% of the genes have expression in the ventral nerve cord primordium P3 , while only 8% of the genes in the data have expression in this region. Similarly, 73% of the genes in cluster 11 have expression in the trunk mesoderm primordium, while only 16% of the genes in the data have expression in this region. For the 13–16 stage network, all 12 clusters are enriched for a total of 81 enrichments, a part of which is visualized in Figure 10. Tables S1 and S2 in the supplementary material report the complete enrichment analysis.

#### Triangulation improves quality of result

**Figure 11:**
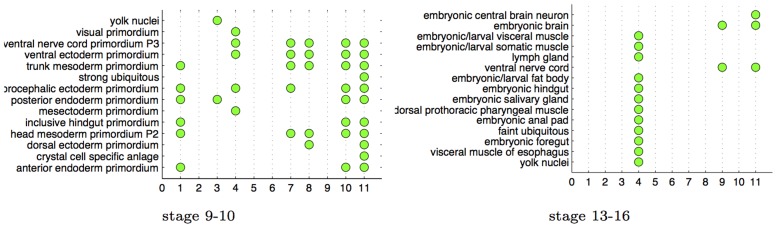
Enrichment analysis on networks learned from SIFT dictionary features instead of triangulation features. The network for development stage 9–10 has only 7 enriched clusters of the 12 clusters in the network. For the stage 13–16 network, only 3 of the 12 clusters are enriched for spatial annotations.

**Figure 12:**
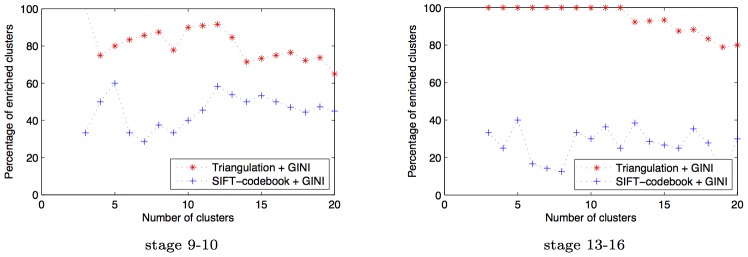
SIFT codebook features do not perform as well as triangulated features on ISH data. Percentage of clusters enriched for spatial annotations in networks predicted by GINI as a function of number of clusters for data from development stage 9–10 and 13–16. As can be seen, using triangulated features produces networks with more enriched clusters than using SIFT-codeword features, independent of the number of clusters selected for the analysis. Further, the enrichment of the GINI network clusters does not significantly vary as the number of clusters are varied.

Previous work on image processing for ISH images has focused on using SIFT features, and constructing a codebook that contain all the embryonic structures that the system is expected to annotate [24]. In this section, we show that triangulation produces more interesting networks over such a SIFT feature representation. We use the 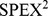 gene expression patterns, and represent them by constructing SIFT features of the expression pattern over a grid. These grid SIFT features are then represented with a codebook of 2000 dictionary features, as described in [24]. We then use these dictionary features instead of the triangulated features to learn the GINI network. Figure 11 shows that the resulting networks are not as richly enriched as the ones derived from the triangulation features in Figure 10. The total number of enrichments in the SIFT codebook network is 42 and 21 for the 9–10 and 13–16 development stage networks respectively. In contrast, the triangulated GINI networks had 63 and 81 enrichments for the 9–10 and 13–16 stage networks. Figure 12 shows that this result is independent of the number of clusters selected for the analysis, for both triangulated networks as well as SIFT codebook networks.

#### Sensitivity to the tuning parameter 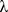


**Figure 13:**
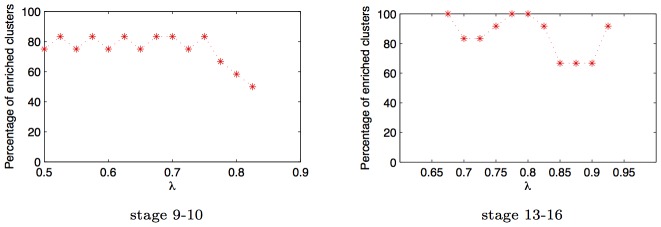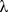 tuning. Percentage of clusters enriched for spatial annotations in networks predicted by GINI as a function of tuning parameter 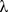 for data from development stage 9–10 and 13–16. As we increase 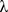, the number of edges predicted in the network decrease, however, the enrichment of the different clusters stays almost constant. Thus, qualitative analysis of the network does not seem to be sensitive to choice of 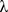.

Supplementary Figure S1 shows how the number of edges in the network decreases as the tuning parameter(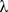) of the GINI algorithm increases. To confirm that the enrichment results are not sensitive to the choice of 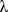, we obtained 21 predicted networks by varying the 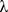 value uniformly from 0.5 to 1. For each network, we repeated the clustering and enrichment analysis, and found that the enrichment for term annotations is not highly sensitive to choice of 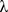 ( Figure 13). The enrichment results are also not dependent on the number of clusters - we get high enrichment, independent of the number of clusters chosen while running the clustering algorithm (Figure 12).

### Comparison with microarray network

**Figure 14:**
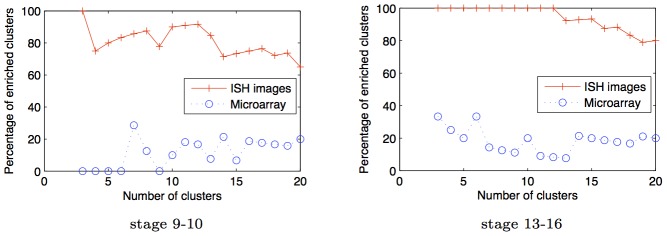
Microarray v/s ISH data. The percentage of clusters that are enriched for spatial term annotations using networks learned from ISH and microarray data.

We learn a network from microarray data collected by the BDGP project over 12 time points in embryonic development [16], over the same genes that are being studied in the 9–10 and 13–16 networks, using covariance between the microarray expression as the kernel. We find that the overlap in edges between the 2 networks is very small, only 1% of the edges are common to both networks. If we assume that spatial expression annotations are a proxy for functional enrichment, then we can check if the microarray network is enriched for the spatial annotation terms. Figure 14 shows that the percentage of enriched clusters in the microarray network is small, independent of the number of clusters analyzed.

We can also test functional GO enrichment of the hubs of the network. Table 2 shows that the hubs of the microarray network for stage 13–16 are enriched for only a single function, where 4 of the 145 hub genes are involved in the “aromatic compound catabolic process”, while the microarray data network for stage 9–10 has no enrichments.

**Table 2:** GO functional analysis for the gene hubs of the microarray network.

Stage	Gene Ontology term	Hub frequency	Genome frequency	P-value
13–16	aromatic compound catabolic process	4 of 145 genes, 2.8%	6 of 3213 genes, 0.2%	0.01841

GO functional analysis for the gene hubs of the microarray networks learned for genes with images in the 13–16 development stage. No enriched terms were found for the microarray network constructed on genes from the 9–10 stage.

Thus, we find that the network learned from ISH images is clearly different from a network learned from microarray data. The ISH image network is enriched for spatial annotation terms, as well as functional enrichment of the hubs of the network, which does not hold true for the microarray network. This suggests that analyzing ISH images could support different scientific conclusions, which should be studied in greater detail.

## Discussion

GINI predicts gene interaction networks by analyzing Drosophila embryo ISH images. While the experiments above have been reported on the ISH data from BDGP, the GINI algorithm can be applied to all image data, by suitably modifying only the image processing 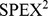 pipeline. Using synthetic and image data, we establish that GINI fits the ISH data well, with low error residues, and that it can learn the true network correctly even if the data is not completely i.i.d. The analysis of the BDGP data shows that the hubs of the predicted gene interaction network are enriched for essential cellular functions, and that different regions of the interaction network are enriched for different combinations of annotation terms describing the gene expression. Thus, the predicted gene interaction network is capturing essential spatial and functional information about the expression pattern of the genes. We found that the gene interaction network learned from ISH images differs significantly from a network learned from microarray data.

The current work focuses on extracting gene networks from spatial data. The next step is combining information from multiple time stages to improve predictions, thus learning spatial-temporal gene networks. The problem of time-varying networks has been studied extensively for microarray data, by using different statistical penalties to estimate the network. For example, Ahmed et. al. [22] construct time varying networks by using a temporally smoothed 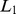-regularized logistic regression formulation, while Danaher et. al. [53] propose a fused lasso and group lasso based approach to combine information across time. Extensions of such algorithms for image data require stronger assumptions on data quality, such as having the same number of genes and image quality across time. Further, certain development stages may be less informative than others; for example, very few genes are active at development stage 1–3, and expression data from this stage is not as informative as expression data from development stage 13–16, when the embryo is much more mature. Developing algorithms that can account for such variations in data quality, while combining information across time, remains an interesting future direction to explore.

## Supporting Information

Dataset S1
**Networks predicted by GINI for the 9–10 and 13–16 development stages.** For the data at each stage, multiple networks were predicted by varying the tuning parameter 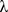, between 0.5 and 1, as described in the paper. The network for each 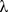 value is stored in a separate file in the dataset, in a format readable by Cytoscape.(BZ2)

Figure S1
**Number of predicted edges versus** 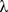. Number of edges predicted by GINI as a function of tuning parameter 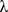 for data from development stage 9–10 and 13–16. As 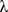 decreases, the number of edges selected in the network increase.(TIFF)

Table S1 **Enrichment analysis for network for development stage 9–10.** For each of the 12 clusters in the GINI network for stage 9–10, the spatial annotation terms for which each cluster is enriched is shown. 11 of the 12 clusters are enriched for at least one spatial annotation.
(PDF)

Table S2 **Enrichment analysis for network for development stage 13–16.** For each of the 12 clusters in the GINI network for stage 13–16, the spatial annotation terms for which each cluster is enriched is shown.
(PDF)
