# Supplementary figures and images for: GINI: From ISH Images to Gene Interaction Networks
Source: PLoS Comput Biol. 2013 Oct 10;9(10):e1003227. doi: 10.1371/journal.pcbi.1003227 (PMC3794902; doi:10.1371/journal.pcbi.1003227)

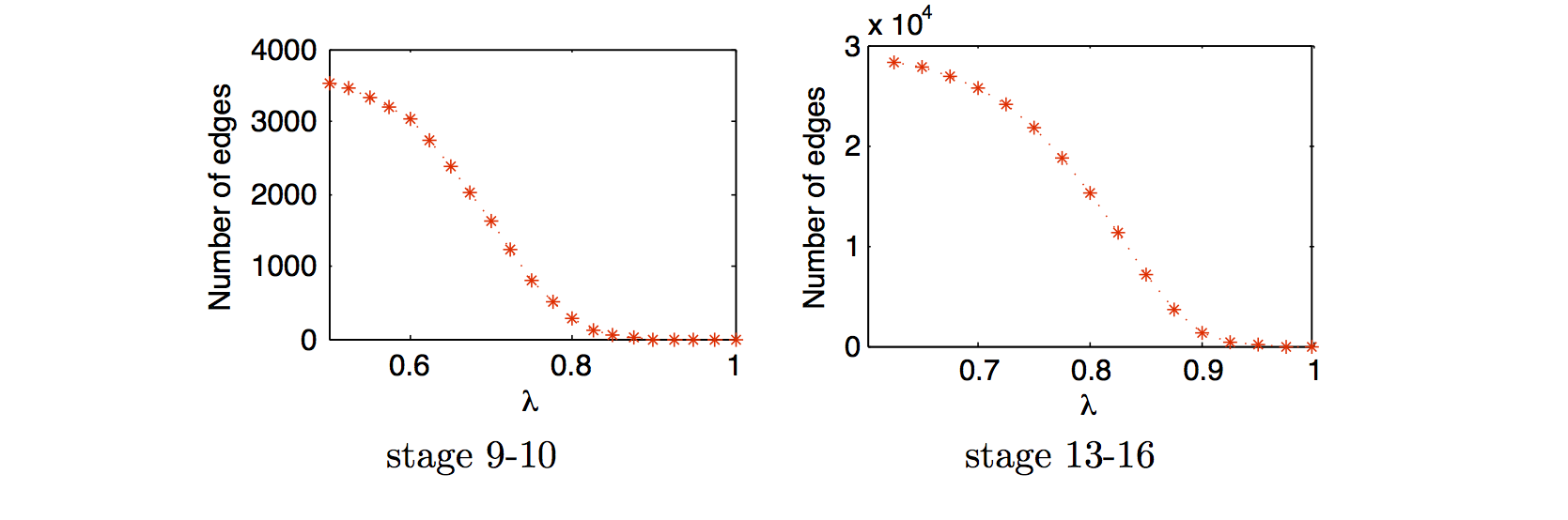

Supplement: Figure S1 — Number of predicted edges versus . Number of edges predicted by GINI as a function of tuning parameter for data from development stage 9–10 and 13–16. As decreases, the number of edges selected in the network increase. (TIFF) [file pcbi.1003227.s002.tiff]
