# Supplementary material for: GINI: From ISH Images to Gene Interaction Networks
Source: PLoS Comput Biol. 2013 Oct 10;9(10):e1003227. doi: 10.1371/journal.pcbi.1003227 (PMC3794902; doi:10.1371/journal.pcbi.1003227)
Supplement: Table S1 — Enrichment analysis for network for development stage 9–10. For each of the 12 clusters in the GINI network for stage 9–10, the spatial annotation terms for which each cluster is enriched is shown. 11 of the 12 clusters are enriched for at least one spatial annotation. (PDF) [file pcbi.1003227.s003.pdf]

Cluster	Gene Ontology term	Cluster frequency	Genome frequency	Corrected P-value
1	procephalic ectoderm primordium	10 of 13 genes, 76.92%	259 of 2609 genes, 9.93%	7.93989e-07
	ventral ectoderm primordium	5 of 13 genes, 38.46%	195 of 2609 genes, 7.47%	0.0199799
	anterior endoderm primordium	7 of 13 genes, 53.85%	361 of 2609 genes, 13.84%	0.0113986
	ventral nerve cord primordium P3	8 of 13 genes, 61.54%	214 of 2609 genes, 8.20%	3.72969e-05
2	procephalic ectoderm primordium	9 of 22 genes, 40.91%	259 of 2609 genes, 9.93%	0.00584779
	visual primordium	3 of 22 genes, 13.64%	38 of 2609 genes, 1.46%	0.0278128
	anterior endoderm primordium	9 of 22 genes, 40.91%	361 of 2609 genes, 13.84%	0.0182396
	trunk mesoderm primordium	10 of 22 genes, 45.45%	416 of 2609 genes, 15.94%	0.0182396
	ventral nerve cord primordium P3	7 of 22 genes, 31.82%	214 of 2609 genes, 8.20%	0.0182396
	head mesoderm primordium P2	9 of 22 genes, 40.91%	383 of 2609 genes, 14.68%	0.0223705
4	dorsal ectoderm primordium	4 of 11 genes, 36.36%	107 of 2609 genes, 4.10%	0.0108335
	procephalic ectoderm primordium	5 of 11 genes, 45.45%	259 of 2609 genes, 9.93%	0.0298304
	ventral ectoderm primordium	5 of 11 genes, 45.45%	195 of 2609 genes, 7.47%	0.0108335
	yolk nuclei	4 of 11 genes, 36.36%	85 of 2609 genes, 3.26%	0.0108335
5	procephalic ectoderm primordium	5 of 7 genes, 71.43%	259 of 2609 genes, 9.93%	0.00759191
	ventral nerve cord primordium P3	4 of 7 genes, 57.14%	214 of 2609 genes, 8.20%	0.0290861
6	posterior endoderm primordium	10 of 12 genes, 83.33%	380 of 2609 genes, 14.56%	8.92704e-06
	inclusive hindgut primordium	5 of 12 genes, 41.67%	185 of 2609 genes, 7.09%	0.00821199
	anterior endoderm primordium	6 of 12 genes, 50.00%	361 of 2609 genes, 13.84%	0.0195944
	trunk mesoderm primordium	6 of 12 genes, 50.00%	416 of 2609 genes, 15.94%	0.0357272
	head mesoderm primordium P2	7 of 12 genes, 58.33%	383 of 2609 genes, 14.68%	0.00651296
	yolk nuclei	5 of 12 genes, 41.67%	85 of 2609 genes, 3.26%	0.000496687
	amnioserosa primordium	3 of 12 genes, 25.00%	48 of 2609 genes, 1.84%	0.0087775
	dorsal epidermis anlage	2 of 12 genes, 16.67%	8 of 2609 genes, 0.31%	0.00651296
7	inclusive hindgut primordium	6 of 10 genes, 60.00%	185 of 2609 genes, 7.09%	0.000892727
	salivary gland duct specific anlage	1 of 10 genes, 10.00%	1 of 2609 genes, 0.04%	0.0352626
	anterior endoderm primordium	6 of 10 genes, 60.00%	361 of 2609 genes, 13.84%	0.0176054
	posterior endoderm primordium	6 of 10 genes, 60.00%	380 of 2609 genes, 14.56%	0.0176054
	trunk mesoderm primordium	6 of 10 genes, 60.00%	416 of 2609 genes, 15.94%	0.0216104
8	procephalic ectoderm primordium	10 of 13 genes, 76.92%	259 of 2609 genes, 9.93%	3.96995e-07
	ventral ectoderm primordium	5 of 13 genes, 38.46%	195 of 2609 genes, 7.47%	0.0205037
	trunk mesoderm primordium	7 of 13 genes, 53.85%	416 of 2609 genes, 15.94%	0.0205037
	ventral nerve cord primordium P3	12 of 13 genes, 92.31%	214 of 2609 genes, 8.20%	3.85669e-11
9	trunk mesoderm primordium	10 of 16 genes, 62.50%	416 of 2609 genes, 15.94%	0.00142784
	ventral ectoderm primordium	6 of 16 genes, 37.50%	195 of 2609 genes, 7.47%	0.0105114
	ventral nerve cord primordium P3	6 of 16 genes, 37.50%	214 of 2609 genes, 8.20%	0.0129817
	head mesoderm primordium P2	9 of 16 genes, 56.25%	383 of 2609 genes, 14.68%	0.00292224
10	head mesoderm primordium P2	9 of 13 genes, 69.23%	383 of 2609 genes, 14.68%	0.000552164
	anterior endoderm primordium	8 of 13 genes, 61.54%	361 of 2609 genes, 13.84%	0.00190482
	posterior endoderm primordium	8 of 13 genes, 61.54%	380 of 2609 genes, 14.56%	0.00190482
	trunk mesoderm primordium	8 of 13 genes, 61.54%	416 of 2609 genes, 15.94%	0.00276461
	crystal cell specific anlage	2 of 13 genes, 15.38%	12 of 2609 genes, 0.46%	0.0112788
	yolk nuclei	3 of 13 genes, 23.08%	85 of 2609 genes, 3.26%	0.0495212
	amnioserosa primordium	3 of 13 genes, 23.08%	48 of 2609 genes, 1.84%	0.0112788
11	head mesoderm primordium P2	48 of 67 genes, 71.64%	383 of 2609 genes, 14.68%	5.84695e-25
	trunk mesoderm primordium	49 of 67 genes, 73.13%	416 of 2609 genes, 15.94%	9.8449e-25
	inclusive hindgut primordium	22 of 67 genes, 32.84%	185 of 2609 genes, 7.09%	2.31777e-09
	anterior endoderm primordium	44 of 67 genes, 65.67%	361 of 2609 genes, 13.84%	6.2578e-22
	posterior endoderm primordium	43 of 67 genes, 64.18%	380 of 2609 genes, 14.56%	5.15659e-20
	procephalic ectoderm primordium	14 of 67 genes, 20.90%	259 of 2609 genes, 9.93%	0.0276785
	foregut primordium	7 of 67 genes, 10.45%	85 of 2609 genes, 3.26%	0.0276785
	ventral nerve cord primordium P3	20 of 67 genes, 29.85%	214 of 2609 genes, 8.20%	1.10392e-06
	central brain primordium P3	5 of 67 genes, 7.46%	41 of 2609 genes, 1.57%	0.0231197
12	trunk mesoderm primordium	10 of 17 genes, 58.82%	416 of 2609 genes, 15.94%	0.00297849
	dorsal ectoderm primordium	5 of 17 genes, 29.41%	107 of 2609 genes, 4.10%	0.0101394
	inclusive hindgut primordium	5 of 17 genes, 29.41%	185 of 2609 genes, 7.09%	0.0266064
	procephalic ectoderm primordium	6 of 17 genes, 35.29%	259 of 2609 genes, 9.93%	0.0250286
	ventral ectoderm primordium	6 of 17 genes, 35.29%	195 of 2609 genes, 7.47%	0.01108
	anterior endoderm primordium	8 of 17 genes, 47.06%	361 of 2609 genes, 13.84%	0.01108
	posterior endoderm primordium	8 of 17 genes, 47.06%	380 of 2609 genes, 14.56%	0.01108
	head mesoderm primordium P2	8 of 17 genes, 47.06%	383 of 2609 genes, 14.68%	0.01108
	strong ubiquitous	2 of 17 genes, 11.76%	13 of 2609 genes, 0.50%	0.0196426
