# Supplementary material for: GINI: From ISH Images to Gene Interaction Networks
Source: PLoS Comput Biol. 2013 Oct 10;9(10):e1003227. doi: 10.1371/journal.pcbi.1003227 (PMC3794902; doi:10.1371/journal.pcbi.1003227)
Supplement: Table S2 — Enrichment analysis for network for development stage 13–16. For each of the 12 clusters in the GINI network for stage 13–16, the spatial annotation terms for which each cluster is enriched is shown. (PDF) [file pcbi.1003227.s004.pdf]

| Cluster | Gene Ontology term | Cluster frequency | Genome frequency | Corrected P-value |
|---|---|---|---|---|
| 1 | embryonic brain | 87 of 103 genes, 84.47% | 705 of 3258 genes, 21.64% | 1.55817e-41 |
| | embryonic central nervous system | 25 of 103 genes, 24.27% | 244 of 3258 genes, 7.49% | 1.23497e-06 |
| | ventral nerve cord | 87 of 103 genes, 84.47% | 722 of 3258 genes, 22.16% | 6.38388e-41 |
| | sensory nervous system primordium | 10 of 103 genes, 9.71% | 88 of 3258 genes, 2.70% | 0.00481947 |
| | ventral sensory complex primordium | 21 of 103 genes, 20.39% | 123 of 3258 genes, 3.78% | 2.89248e-09 |
| | embryonic midgut | 52 of 103 genes, 50.49% | 943 of 3258 genes, 28.94% | 3.97885e-05 |
| | ventral midline | 15 of 103 genes, 14.56% | 149 of 3258 genes, 4.57% | 0.000724672 |
| | dorsal/lateral sensory complexes | 20 of 103 genes, 19.42% | 114 of 3258 genes, 3.50% | 3.93463e-09 |
| | sensory system head | 23 of 103 genes, 22.33% | 167 of 3258 genes, 5.13% | 1.73336e-08 |
| 2 | embryonic dorsal epidermis | 9 of 9 genes, 100.00% | 511 of 3258 genes, 15.68% | 6.33068e-06 |
| | embryonic head epidermis | 8 of 9 genes, 88.89% | 351 of 3258 genes, 10.77% | 8.05947e-06 |
| | embryonic ventral epidermis | 7 of 9 genes, 77.78% | 472 of 3258 genes, 14.49% | 0.0010393 |
| | embryonic dorsal apodeme | 2 of 9 genes, 22.22% | 28 of 3258 genes, 0.86% | 0.0361365 |
| | embryonic epipharynx | 4 of 9 genes, 44.44% | 246 of 3258 genes, 7.55% | 0.0383597 |
| | embryonic hypopharynx | 4 of 9 genes, 44.44% | 270 of 3258 genes, 8.29% | 0.0486715 |
| | embryonic ventral apodeme | 2 of 9 genes, 22.22% | 26 of 3258 genes, 0.80% | 0.0356104 |
| | embryonic foregut | 6 of 9 genes, 66.67% | 290 of 3258 genes, 8.90% | 0.0010393 |
| | embryonic hindgut | 6 of 9 genes, 66.67% | 578 of 3258 genes, 17.74% | 0.0306731 |
| | embryonic/larval tracheal system | 4 of 9 genes, 44.44% | 199 of 3258 genes, 6.11% | 0.0306731 |
| 3 | embryonic salivary gland body | 7 of 13 genes, 53.85% | 85 of 3258 genes, 2.61% | 1.13596e-06 |
| | embryonic salivary gland | 5 of 13 genes, 38.46% | 104 of 3258 genes, 3.19% | 0.00184931 |
| 4 | yolk nuclei | 23 of 27 genes, 85.19% | 170 of 3258 genes, 5.22% | 1.24293e-24 |
| 5 | embryonic brain | 12 of 12 genes, 100.00% | 705 of 3258 genes, 21.64% | 7.63985e-07 |
| | embryonic central nervous system | 6 of 12 genes, 50.00% | 244 of 3258 genes, 7.49% | 0.00407528 |
| | ventral nerve cord | 12 of 12 genes, 100.00% | 722 of 3258 genes, 22.16% | 7.63985e-07 |
| | sensory system head | 5 of 12 genes, 41.67% | 167 of 3258 genes, 5.13% | 0.00575847 |
| 6 | embryonic midgut | 18 of 21 genes, 85.71% | 943 of 3258 genes, 28.94% | 1.09435e-05 |
| 7 | ventral nerve cord | 14 of 14 genes, 100.00% | 722 of 3258 genes, 22.16% | 3.65112e-08 |
| | embryonic brain | 14 of 14 genes, 100.00% | 705 of 3258 genes, 21.64% | 3.65112e-08 |
| 8 | embryonic brain | 7 of 7 genes, 100.00% | 705 of 3258 genes, 21.64% | 0.00150103 |
| | embryonic central nervous system | 4 of 7 genes, 57.14% | 244 of 3258 genes, 7.49% | 0.0349876 |
| | ventral nerve cord | 7 of 7 genes, 100.00% | 722 of 3258 genes, 22.16% | 0.00150103 |
| 9 | embryonic hindgut | 14 of 23 genes, 60.87% | 578 of 3258 genes, 17.74% | 0.00052391 |
| | embryonic/larval tracheal system | 7 of 23 genes, 30.43% | 199 of 3258 genes, 6.11% | 0.00707219 |
| | embryonic dorsal epidermis | 11 of 23 genes, 47.83% | 511 of 3258 genes, 15.68% | 0.00707219 |
| | embryonic epipharynx | 7 of 23 genes, 30.43% | 246 of 3258 genes, 7.55% | 0.0161065 |
| | embryonic esophagus | 5 of 23 genes, 21.74% | 111 of 3258 genes, 3.41% | 0.0144353 |
| | embryonic hypopharynx | 9 of 23 genes, 39.13% | 270 of 3258 genes, 8.29% | 0.00273453 |
| | embryonic ventral epidermis | 9 of 23 genes, 39.13% | 472 of 3258 genes, 14.49% | 0.0375182 |
| | embryonic salivary gland body | 4 of 23 genes, 17.39% | 85 of 3258 genes, 2.61% | 0.034015 |
| | embryonic foregut | 9 of 23 genes, 39.13% | 290 of 3258 genes, 8.90% | 0.00322211 |
| | embryonic salivary gland | 5 of 23 genes, 21.74% | 104 of 3258 genes, 3.19% | 0.0124865 |
| 10 | ventral nerve cord | 12 of 12 genes, 100.00% | 722 of 3258 genes, 22.16% | 7.63985e-07 |
| | ventral sensory complex primordium | 5 of 12 genes, 41.67% | 123 of 3258 genes, 3.78% | 0.00132457 |
| | ventral midline | 6 of 12 genes, 50.00% | 149 of 3258 genes, 4.57% | 0.000237634 |
| | embryonic brain | 12 of 12 genes, 100.00% | 705 of 3258 genes, 21.64% | 7.63985e-07 |
| | embryonic central nervous system | 6 of 12 genes, 50.00% | 244 of 3258 genes, 7.49% | 0.00244517 |
| | sensory system head | 4 of 12 genes, 33.33% | 167 of 3258 genes, 5.13% | 0.0398581 |
| | dorsal/lateral sensory complexes | 4 of 12 genes, 33.33% | 114 of 3258 genes, 3.50% | 0.0110463 |
| 11 | embryonic brain | 10 of 10 genes, 100.00% | 705 of 3258 genes, 21.64% | 1.5916e-05 |
| | embryonic central nervous system | 5 of 10 genes, 50.00% | 244 of 3258 genes, 7.49% | 0.0162686 |
| | ventral nerve cord | 10 of 10 genes, 100.00% | 722 of 3258 genes, 22.16% | 1.5916e-05 |
| 12 | embryonic/larval muscle system | 270 of 581 genes, 46.47% | 435 of 3258 genes, 13.35% | 1.17801e-112 |
| | dorsal prothoracic pharyngeal muscle | 200 of 581 genes, 34.42% | 312 of 3258 genes, 9.58% | 1.45332e-82 |
| | embryonic midgut | 388 of 581 genes, 66.78% | 943 of 3258 genes, 28.94% | 9.0204e-98 |
| | embryonic hindgut | 235 of 581 genes, 40.45% | 578 of 3258 genes, 17.74% | 1.33932e-46 |
| | embryonic Malpighian tubule | 109 of 581 genes, 18.76% | 221 of 3258 genes, 6.78% | 1.34134e-27 |
| | embryonic anal pad | 112 of 581 genes, 19.28% | 257 of 3258 genes, 7.89% | 1.32288e-22 |
| | embryonic/larval fat body | 99 of 581 genes, 17.04% | 215 of 3258 genes, 6.60% | 4.56185e-22 |
| | embryonic/larval somatic muscle | 72 of 581 genes, 12.39% | 142 of 3258 genes, 4.36% | 7.97006e-19 |
| | embryonic gastric caecum | 39 of 581 genes, 6.71% | 75 of 3258 genes, 2.30% | 1.26069e-10 |
| | embryonic proventriculus | 59 of 581 genes, 10.15% | 225 of 3258 genes, 6.91% | 0.00506482 |
| | embryonic proventriculus outer layer | 11 of 581 genes, 1.89% | 26 of 3258 genes, 0.80% | 0.0178799 |
| | embryonic dorsal epidermis | 112 of 581 genes, 19.28% | 511 of 3258 genes, 15.68% | 0.0287759 |
| | embryonic ventral epidermis | 104 of 581 genes, 17.90% | 472 of 3258 genes, 14.49% | 0.0319466 |
| | embryonic foregut | 88 of 581 genes, 15.15% | 290 of 3258 genes, 8.90% | 2.86139e-07 |
| | embryonic salivary gland | 38 of 581 genes, 6.54% | 104 of 3258 genes, 3.19% | 2.59444e-05 |
| | crystal cell | 22 of 581 genes, 3.79% | 71 of 3258 genes, 2.18% | 0.0252076 |
| | embryonic/larval visceral muscle | 46 of 581 genes, 7.92% | 107 of 3258 genes, 3.28% | 6.67701e-09 |
| | circular visceral muscle fibers | 5 of 581 genes, 0.86% | 9 of 3258 genes, 0.28% | 0.0496811 |
| | longitudinal visceral muscle fibers | 10 of 581 genes, 1.72% | 15 of 3258 genes, 0.46% | 0.000299961 |
| | embryonic/larval dorsal vessel | 19 of 581 genes, 3.27% | 59 of 3258 genes, 1.81% | 0.0270534 |
| | embryonic/larval garland cell | 31 of 581 genes, 5.34% | 112 of 3258 genes, 3.44% | 0.0287759 |
| | lymph gland | 36 of 581 genes, 6.20% | 80 of 3258 genes, 2.46% | 1.12049e-07 |
| | germ cell | 18 of 581 genes, 3.10% | 57 of 3258 genes, 1.75% | 0.0343357 |
| | gonad | 41 of 581 genes, 7.06% | 160 of 3258 genes, 4.91% | 0.0328045 |
| | plasmatocytes | 23 of 581 genes, 3.96% | 73 of 3258 genes, 2.24% | 0.0178799 |
| | yolk nuclei | 46 of 581 genes, 7.92% | 170 of 3258 genes, 5.22% | 0.00920771 |
| | visceral muscle of esophagus | 10 of 581 genes, 1.72% | 16 of 3258 genes, 0.49% | 0.000630892 |
| | faint ubiquitous | 90 of 581 genes, 15.49% | 236 of 3258 genes, 7.24% | 1.46814e-13 |
